# Clinical and genetic analysis of Christianson syndrome caused by variant of *SLC9A6*: case report and literature review

**DOI:** 10.3389/fneur.2023.1152696

**Published:** 2023-05-05

**Authors:** Yan Dong, Ruofei Lian, Liang Jin, Shichao Zhao, Wenpeng Tao, Lijun Wang, Mengchun Li, Tianming Jia, Xuejing Chen, Shushi Cao

**Affiliations:** ^1^Department of Pediatrics, The Third Affiliated Hospital of Zhengzhou University, Zhengzhou, China; ^2^Henan Key Laboratory of Child Brain Injury and Henan Pediatric Clinical Research Center, The Third Affiliated Hospital and Institute of Neuroscience, Zhengzhou, China

**Keywords:** *SLC9A6* gene variant, Christianson syndrome, epilepsy, global developmental delay, whole-exome sequencing, genetics

## Abstract

**Background:**

Intellectual disability, X-linked, syndromic, Christianson type (MRXSCH, OMIM: 300243)—known as Christianson syndrome (CS)—is characterized by microcephaly, epilepsy, ataxia, and absence of verbal language ability. CS is attributed to mutations in the solute carrier family 9 member A6 gene (*SLC9A6*).

**Materials and methods:**

This study reports the case of a boy 1 year and 3 months of age who was diagnosed with CS in our department. Genetic etiology was determined by whole-exome sequencing, and a minigene splicing assay was used to verify whether the mutation affected splicing. A literature review of CS cases was conducted and the clinical and genetic features were summarized.

**Results:**

The main clinical manifestations of CS include seizures, developmental regression, and exceptional facial features. Whole-exome sequencing revealed a *de novo* splice variant in intron 11 (c.1366 + 1G > C) of *SLC9A6*. The mutation produced two abnormal mRNA products (verified by a minigene splicing assay), resulting in the formation of truncated protein. A total of 95 CS cases were identified in the literature, with various symptoms, such as delayed intellectual development (95/95, 100.00%), epilepsy (87/88, 98.86%), and absent verbal language (75/83, 90.36%). At least 50 pathogenic variants of *SLC9A6* have been identified, with the highest frequency observed in exon 12.

**Conclusion:**

Our patient is the first case with the c.1366 + 1G > C variant of *SLC9A6* in CS. The summary of known cases can serve as a reference for analyzing the mutation spectrum and pathogenesis of CS.

## 1. Introduction

Intellectual disability, X-linked, syndromic, Christianson type (MRXSCH, OMIM:300243)—known as Christianson syndrome (CS)—is an X-linked neurodevelopmental syndrome that occurs in males. CS is characterized by progressive developmental delay, developmental regression, and intellectual disability. Patients may present with absent verbal language, autistic symptoms, craniofacial dysmorphism, ataxia, oculomotor palsy, and different types of early-onset seizure; exhibit delays in height, weight, and postnatal brain growth; and may not be able to walk even at 10 years of age (1–4). The electroencephalography (EEG) profiles of patients with CS are characterized by a fast (10–14 Hz) background rhythm, with sharp and slow waves in the frontopolar, frontal, anterior temporal, and frontal midline regions (5). Carrier females have been described as having learning difficulties with mild-to-moderate intellectual disability, behavioral issues, and psychiatric illnesses (6).

Christianson syndrome has a prevalence of 1:16,000–1:100,000 (7). Pathogenic mutations in the solute carrier family 9-member A6 gene (*SLC9A6*), encoding Na+/H+ exchanger protein member 6 (NHE6), have been associated with CS and autism spectrum disorders (5). From the first description of CS by Christianson et al. (8) to October 2022, 95 cases have been reported, of which five are from China. In this case report, a *de novo* mutation of *SLC9A6* is described in a patient with CS, with details of the specific clinical course. Additionally, a literature review of reported cases of CS is presented.

## 2. Materials and methods

### 2.1. Patient and ethics

In January 2022, a Chinese boy aged 1 year and 3 months was admitted to the Department of Pediatric Neurology at the Third Affiliated Hospital of Zhengzhou University with drug-resistant seizures for 2 months. Written informed consent was obtained from the parents for publication of data included in this article.

### 2.2. Whole exome sequencing

#### 2.2.1. Extraction of genomic DNA and sequence analysis

For extraction of genomic DNA, 2 mL of peripheral blood (with anticoagulant EDTA) was collected from the patient, his parents, and two elder brothers. A blood genomic DNA extraction kit (Kangwei Century, Shanghai, China) was used for DNA extraction, according to the manufacturer’s protocol. After quality control and library establishment was complete, DNA fragments in the target region were enriched and a whole exon library was constructed. The probes were captured by xGen Exome Research Panel vl.0 (IDT Company, United States). An Illumina NovaSeq 6000 series sequencer (Illumina, San Diego, CA, United States) was used for high-throughput sequencing. After quality control was complete, off-machine data were compared with the human reference genome (GRCh37/hg19) using Burrows-Wheeler Aligner software (version 0.59). Genome Analysis ToolKit software (version 4.0.4.0) was used to filter and screen the detected SNPs and indels to obtain high-quality, reliable variants.

#### 2.2.2. Mutation confirmed by sanger sequencing

Primers were designed for candidate mutation sites, and PCR amplification was performed to verify mutations and short fragment deletions or insertion in positive sites. Up- and downstream primer sequences were 5′-AGGTGCTCACCAAATTGGAC-3′ and 5′-CAGCTACTTGGGAGGCTGAG-3′, respectively. The PCR program consisted of 35 cycles of denaturation, annealing, and extension. Takara Ex Taq (No. RR001A, Takara Bio, Inc., Otsu, Japan) was used for amplification. The conditions for PCR are presented in Supplementary Table 1. PCR products were sequenced using ABI 3730XL (ABI, Carlsbad, CA, United States), analyzed with DNASTAR software (DNASTAR, Inc., Madison, United States), and compared with the mRNA template (SLC9A6: NM_001042537.2).

### 2.3. Minigene splicing assay

The minigene plasmid was designed to insert between exons 10 and 12 of *SLC9A6*. The primer sequences of amplified gDNA are shown in Supplementary Table 2. The 5′ end of intron 10 (with the 338-bp sequence) was joined with the 3′ end of intron 10 (with the 364-bp sequence) to form intron 10. The amplified products were cloned into the pMini-CopGFP vector (Hitrobio Biotechnology, Beijing, China), digested with restriction enzymes 5′-BamHI/3′-XhoI using ClonExpress II One Step Cloning Kit (Vazyme, Nanjing, China). The mutant plasmid was generated by site-directed mutagenesis of the wild-type plasmid using *SLC9A6*-MUT-F/R primers. The wild-type and variant minigene plasmids, verified by Sanger sequencing, were transiently transfected into human embryonic kidney 293 T cells using Lipofectamine 2000 (Invitrogen, Carlsbad, CA, United States). After 48 h, total RNA was extracted from cells using TRIzol reagent (Cowin Biotech, Jiangsu, China). Primers were designed (MiniRT-F and *SLC9A6*-RT-R, see Supplementary Table 2) for reverse transcription-PCR (RT-PCR) followed by Sanger sequencing. Finally, ExPASy Translate^1^ was used to translate nucleotide sequences into protein sequences, to analyze the effect of the mutations on protein sequence.

### 2.4. Literature review and statistics

**Figure 1:**
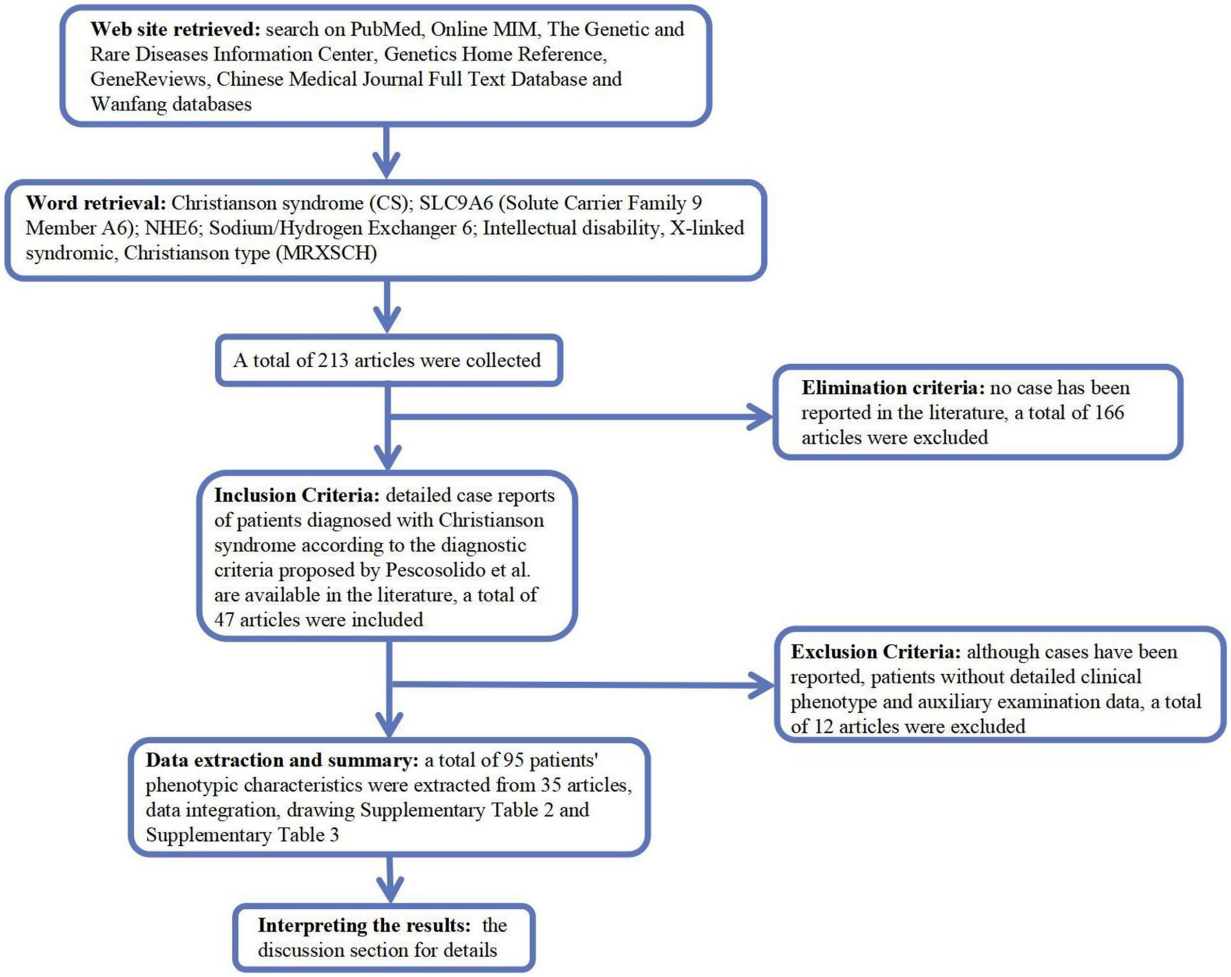
A flow-chart of the study.

We searched PubMed,^2^ Online MIM (OMIM; https://omim.org/), Genetic and Rare Diseases Information Center,^3^ Genetics Home Reference,^4^ GeneReviews,^5^ Chinese Medical Journal Full Text Database,^6^ and Wanfang (Chinese, http://www.wanfangdata.com.cn/) for Christianson syndrome (CS), solute carrier family 9 member A6 (*SLC9A6*), Na+/H+ exchanger 6 (NHE6), intellectual disability, X-linked, syndromic, and Christianson type (MRXSCH; Figure 1).

## 3. Results

### 3.1. Case report

#### 3.1.1. Clinical history

**Figure 2:**
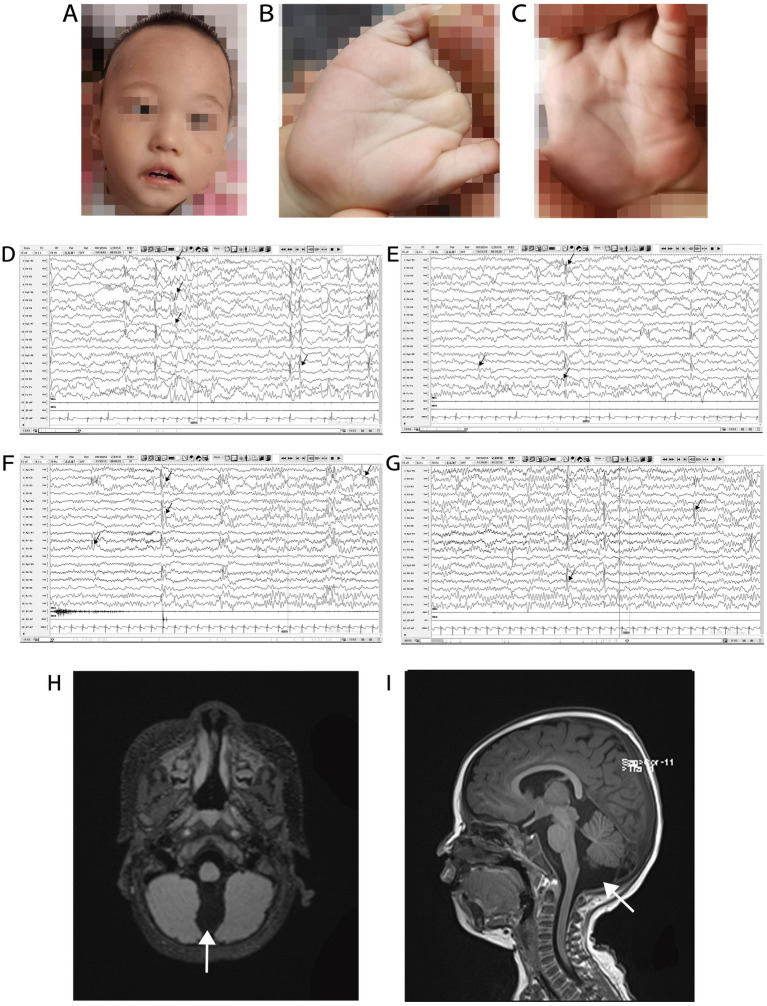
Phenotype, VEEG, and brain MRI of a patient with Christianson syndrome. Facial features included long forehead, wide eye spacing, and convergent strabismus **(A)**. Heterogeneous palm prints **(B,C)**. VEEG from February 16, 2023, when the patient was 2 years and 4 months of age (**D–G**, the black arrow indicates multifocal spike and wave complexes and sharp waves). Brain MRI from December 12, 2021, when the patient was 1 year and 2 months of age (**H** and **I**, the white arrows indicates a prominent cisterna magna). MRI, magnetic resonance imaging; VEEG, video electroencephalograph.

The patient was delivered at term by cesarean section because of placental abruption. The patient lifted his head at 3 months of age, sat up at 6 months of age, and could walk with assistance before the onset of symptoms. In November 2021, at the age of 1 year and 1 month, the patient had generalized tonic–clonic seizure with no apparent cause, which stopped spontaneously from 10 s to 3 min. The seizures continued at a frequency of 2–7 times per day. Since the onset of seizures, the patient has regressed developmentally and is now only capable of four-point support. He is unable to talk, sit, crawl, or stand-up unaided. Physical examination at admission in January 2022 showed that the patient was 80-cm tall (0–1 SD), weighed 9 kg (−2 to −1 SD), and had a head circumference of 42.8 cm (−2 to −1 SD). He had a poor response to stimulation, with a closed fontanelle, long forehead, wide eye spacing, convergent strabismus, and heterogeneous left-hand palm print (Figures 2A–C). The patient had low muscle tone in all limbs. All other physical examinations were normal.

#### 3.1.2. Auxiliary examination

Routine blood examination and blood biochemical indices were normal. The patient’s karyotype and electromyography of the extremities were normal. The ambulatory electrocardiogram (AECG) of the patient at 1 year and 3 months of age, showed premature ventricular contractions (PVCs), with 5,678 beats (3.53%), two bigeminal beats, and 266 trigeminal beats. The AECG of the patient at 1 year and 7 months of age showed persistent arrhythmia and 5,036 PVCs, with 82 bigeminal beats, 20 trigeminal beats, and ventricular parasystole. Cardiac magnetic resonance imaging (MRI) of the patient at 1 year and 7 months of age showed myocarditis changes in the apical and basal segments of the left ventricle. A video EEG (VEEG) of the patient at 1 year and 6 months of age showed a large number of spikes, slow waves, and alpha rhythm in the anterior head. The VEEG of the patient at 2 years and 4 months of age showed multifocal spike and wave complexes and sharp waves (Figures 2D–G). Brain MRI of the patient at 1 year and 2 months of age revealed a prominent cisterna magna (Figures 2H,I). At this time, Alberta Infant Motor Scale score was 52 (<5%), implying that the patient was developmentally delayed.

#### 3.1.3. Diagnosis, treatment, and clinical follow-up

The patient was diagnosed with CS based on *SLC9A6* mutation and clinical manifestations of developmental delay, intermittent convulsions, and developmental regression for 2 months. Based on the AECG data, the patient was diagnosed with sinus arrhythmia. According to the findings of a study (9), the patient had myocarditis changes on cardiac MRI and arrhythmia, and was therefore, diagnosed with suspected myocarditis.

Since the first occurrence of convulsive seizures on November 30, 2021, the patient was being treated with oxcarbazepine (OXC; 24 mg/kg/day) combined with sodium valproate (VPA; 21 mg/kg/day) at other hospitals; however, these treatments were ineffective. The primary treatment at our hospital consisted of three courses: (1) discontinuation of OXC because of arrhythmia and administration of levetiracetam (LEV) to control seizures; (2) functional training and rehabilitation; and (3) nourishment of the myocardium. After 1 month of treatment, the patient’s muscle strength returned to normal in all four limbs. By the last visit, on February 16, 2023, the patient had occasional nystagmus and was seizure-free for 1 year since the initiation of add-on LEV therapy (40 mg/kg/day), with VPA maintained at 21 mg/kg/day. He was 90 cm tall (−2SD to −1 SD), weighed 12.4 kg (−2 to −1 SD), and could crawl and stand alone. However, he required support for walking and could not pinch objects with the thumb and index finger. He could say words such as “dad” and “mom” and had poor language comprehension ability. Griffiths neurodevelopmental assessment showed that he had global neurodevelopmental retardation at 2 years and 4 months of age in February 2023. The patient’s arrhythmia was improved, and nourishment therapy for the myocardium with oral fructose diphosphate, vitamin C, etc., was continued.

### 3.2. Whole exome sequencing

**Figure 3:**
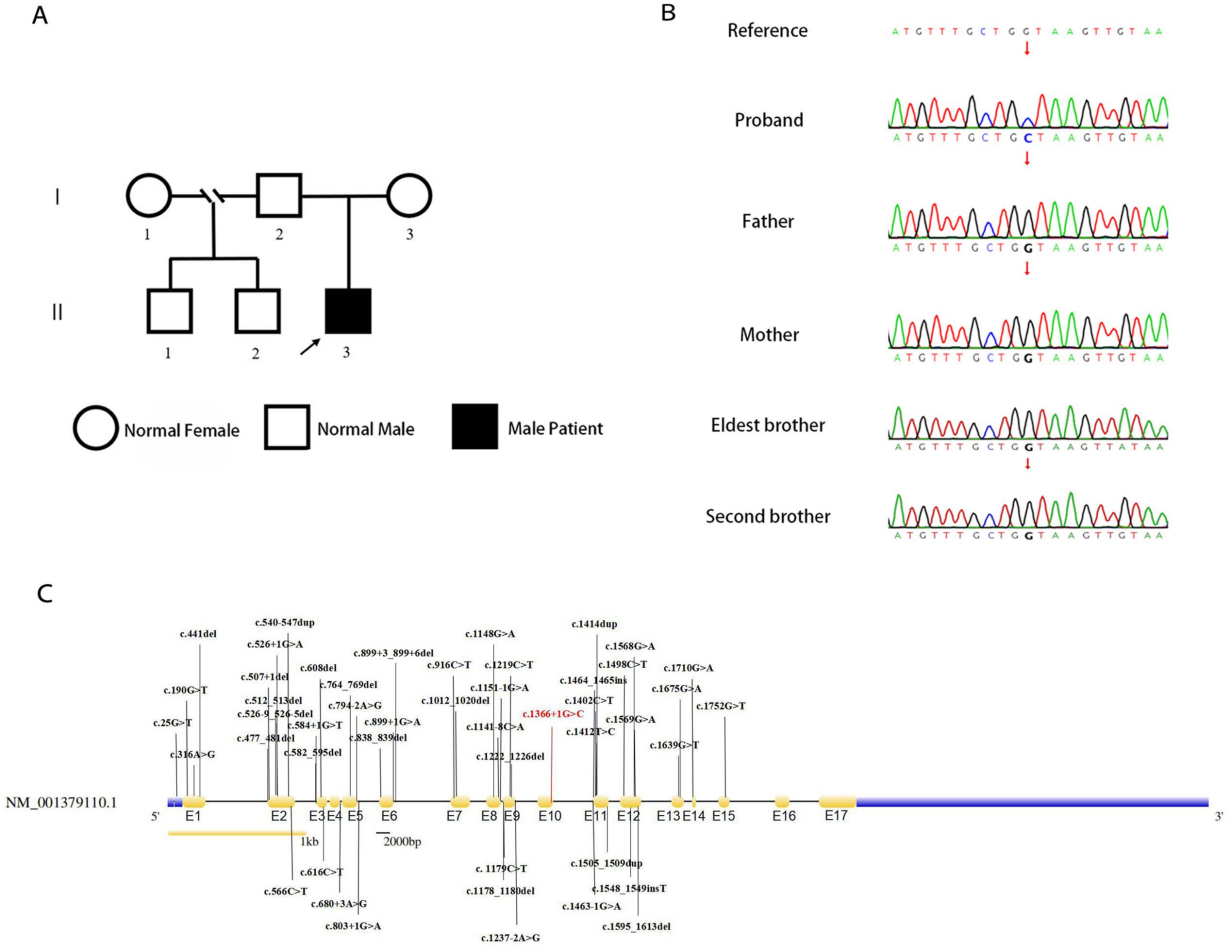
Genealogy map of the patient’s family **(A)**. Sanger sequencing of *SLC9A6* of the patient with Christianson syndrome and his family. The patient had a *de novo* splicing variant in intron 11 (c.1366 + 1G > C) of *SLC9A6* that was not present in his parents and two brothers (arrows; **B**). Structure of *SLC9A6* (searched on NCBI, https://www.ncbi.nlm.nih.gov/gene/). The locations of *SLC9A6* variations are indicated in black (top and bottom); locations of *SLC9A6* variations in the patient reported are in red. The gene has 17 exons (yellow, exons are numbered E1–E17 sequentially; **C**). SLC9A6: solute carrier family 9 member A6.

Genetic analysis of the patient by whole exome sequencing (WES) suggested the presence of a *de novo* splice variant in intron 11 (c.1366 + 1G > C) of *SLC9A6*, which was absent in other family members (Figures 3A,B). The mutation is located in the splice site of intron 11 and is presumed to cause abnormal splicing of *SLC9A6*, which affects exon 12 and leads to CS (PVS1). Moreover, it is a *de novo* variant (PS2). To our knowledge, this is the first case of CS with this variant locus, which is not reported in 1,000 Genomes Project (1000G, https://www.ncbi.nlm.nih.gov/gene/10479) or Genome Aggregation Database (gnomAD, http://www.gnomad-sg.org/; PM2). Pathogenicity analysis performed according to American College of Medical Genetics and Genomics 2019 guidelines (10) indicated that the novel variant was pathogenic (PVS1 + PS2 + PM2).

### 3.3. Pooled analysis of the literature

We summarized 35 studies on CS cases from 1999 to October 2022. The characteristics of the 95 patients with CS and 58 affected families are summarized in Supplementary Table 3. All patients (95/95, 100.00%) had impaired intellectual development. Approximately one-half of the patients had combined developmental regression (25/55, 45.45%), and the remaining had common clinical manifestations, including epilepsy (87/88, 98.86%), absent verbal language despite apparently normal hearing (75/83, 90.36%), microcephaly (61/77, 79.22%), ataxia (49/63, 77.78%), autistic behavior (35/47, 74.47%), ophthalmoplegia (35/49, 71.43%), and hyperkinesia (40/56, 71.43%). Some patients also presented with sleep disorders (17/35, 48.57%), almost one-half of patients exhibited a peculiar constellation of symptoms resembling those encountered in Angelman syndrome (23/50 46.00%), and some had cerebellar atrophy (20/57, 35.09%). A total of 18 adults have been reported, with progressive motor deterioration and ataxia in the affected males beginning in the fourth to fifth decade of life. A study reported two cases, in which unilateral weakness and spasticity developed prior to the loss of ambulation, in a family with all adult patients (11). In the reported cases, no age-related changes in epilepsy phenotypes were found, and there were no significant differences in the phenotypes between adults and children. All identified variant loci of *SLC9A6* are summarized in our study with c.1366 + 1G > C; exon 12 was found to be a hot spot for pathogenic mutations (Figure 3C).

### 3.4. Minigene testing

**Figure 4:**
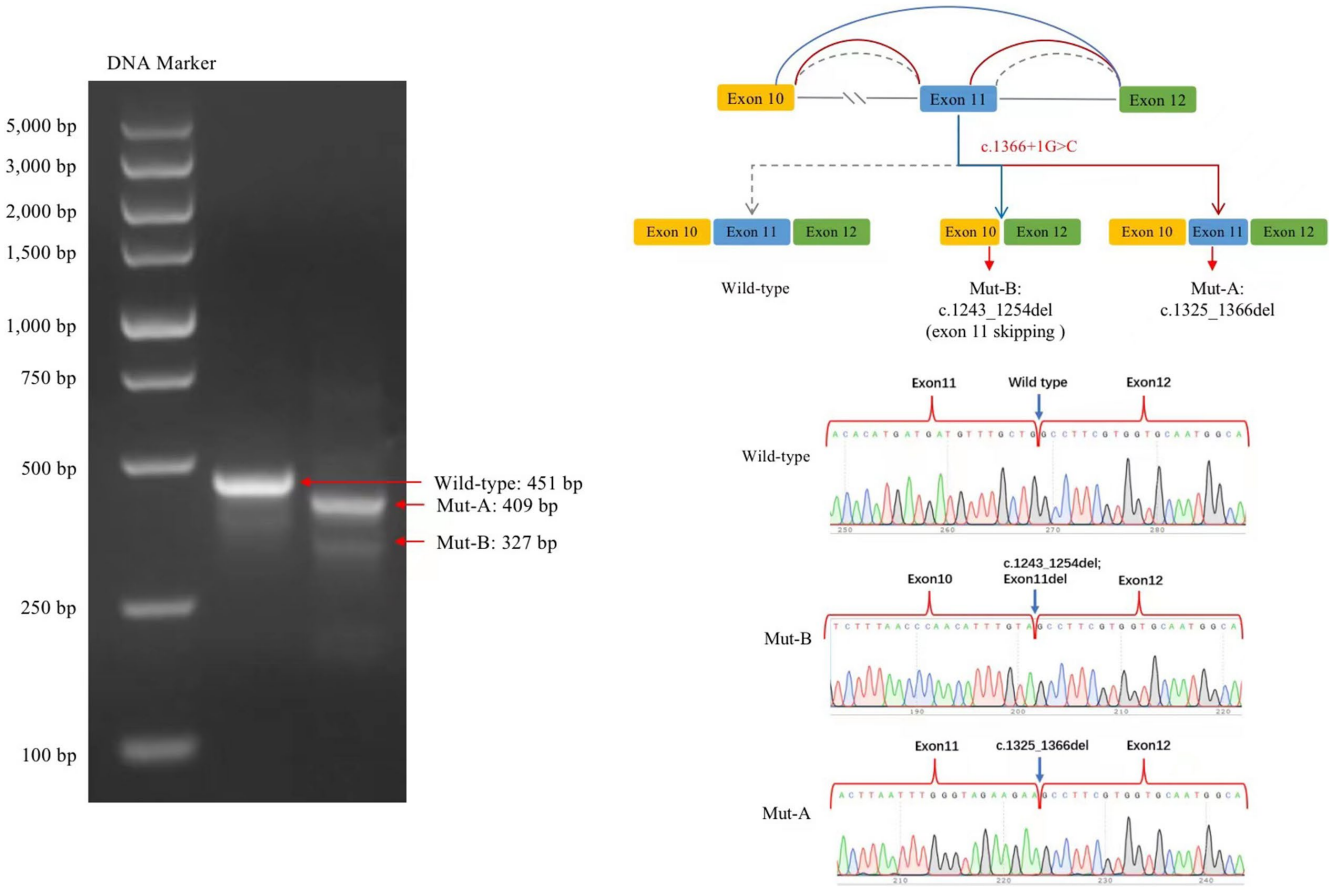
Splicing analysis using a minigene splicing assay. The variant plasmid transcribed two mRNA products: the first was the main splicing form (Mut-A, electrophoretogram bands are brighter than those for Mut-B), with a 42-bp deletion in exon 11. The mRNA was expressed as NM006359.3: c.1325_1366del. The second included a minor splicing form (Mut-B), with a partial deletion of a 12-bp sequence in exon 10 and a complete deletion of a 112-bp sequence in exon 11; the mRNA is expressed as NM006359.3: c.1243_1366del.

The transcribed mRNA sequence from the wild-type plasmid contained complete exon 10–12. The variant plasmid transcribed two mRNA products. The first was the main splicing form, with a 42-bp deletion in exon 11; the mRNA was expressed as NM006359.3: c.1325_1366del (p.Lys443_Gly456del), which may lead to an amino acid shift to form a truncated protein. The second was a minor spliced form, with a partial deletion of 12 bp in exon 10 and a complete deletion of 112 bp in exon 11 (exon 11 skipping); the mRNA was expressed as NM006359.3: 1243_1366del (p.Val415AlafsTer22), which may cause an amino acid shift to form a truncated protein (Figure 4).

## 4. Discussion

Christianson syndrome, which was first reported by Christianson et al. (8), is an X-linked neurodevelopmental disorder, characterized by impaired intellectual development, absent verbal language, early-onset seizures of variable types, and shortened life expectancy. The disease occurs in males, and female carriers may be mildly affected. Diagnostic criteria for CS proposed by Pescosolido et al. (2) include early-childhood onset, nonverbal status, moderate-to-severe intellectual disability, epilepsy, truncal ataxia, postnatal microcephaly and/or attenuation of head circumference growth, and hyperkinetic behavior; secondary symptoms, which are often present, include symptoms of autism and/or Angelman syndrome, eye movement abnormalities, developmental regression (especially loss of independent ambulation after 10 years of age), low weight-for-age, and cerebellar vermis atrophy (2). The findings of a study (6) provided further support for these phenotypes and expanded the phenotypes to suggest a significant risk for various psychiatric disorders, including mood and anxiety disorders, attention deficit hyperactivity disorder, oppositional defiant disorder, and disorders involving psychosis, such as bipolar disorder and schizophrenia.

In 2008, a study identified four mutations in *SLC9A6* in different families that altered the function of NHE6 (12). A total of three fragment deletions of the X chromosome and 47 pathogenic mutations were identified, of which 13 were intron splice site variants; *de novo* chromosomes or mutations accounted for 44.00% (22/50; Figure 3C). Lizarraga et al. demonstrated that pathogenic mutations lead to loss of protein function through various mechanisms—most mutations led to loss of mRNA due to nonsense-mediated mRNA decay (13). The exon mutation profile shows that exon 12 is a hot spot for pathogenic mutations and harbors 43.8% of all variants (14). A 3-year-old Chinese patient with CS had a splicing mutation (c.1463-1G > A) in *SLC9A6*, which was verified by *in vitro* transcription. This intron 11 mutation caused exon 12 skipping. The patient presented with seizures, intellectual disability, microcephaly, feeding difficulties, hyperkinesia, ataxia, and cerebellar atrophy (14). In this report, the mutation identified is a classical splice site mutation in intron 11. Plasmids with a splice variant (c.1366 + 1G > C) of *SLC9A6* was constructed using a minigene splicing assay to transcribe the two mRNA products. The main splicing forms included a partial deletion of 42 bp in exon 11, which may lead to the formation of a truncated protein. A minor splicing form includes a partial deletion of 12 bp in exon 10 and exon 11 skipping, which may lead to an amino acid shift to form a truncated protein. This study speculates that this mutation results in the aberrant splicing of *SLC9A6*, forming truncated proteins and contributing to CS development. However, the main clinical phenotypes of the patient reported in this study were seizures and developmental regression, which were milder than those reported for the 3-year-old Chinese patient with CS, and no genotype–phenotype correlations were observed in this study.

The NHE protein family has nine members. NHE1–5 are present in the cytoplasmic membrane, whereas NHE6–9 are present in organelle membranes. NHE proteins have an N-terminal domain of 10–12 transmembrane helices and a hydrophilic C-terminal domain, which is regulatory. NHE6, a dodecameric transmembrane protein, is localized in early recycling endosomes, but not in lysosomes, and transiently appears on the plasma membrane (15). Overexpression of NHE isoforms increases the luminal pH of the compartment in which the NHE is located; NHE6 changes the early endosomal membrane endoluminal pH from slightly acidic to neutral. Thus, a possible consequence of NHE6 inactivation is a decrease in pH of the endosomal recycling compartment. NHE6 overexpression reduces monovalent ion content, which may affect protein folding and transport (15, 16). Neurons with *SLC9A6* knockout had fewer lysosomes in the cytoplasm and reduced activity of lysosomal proteases; moreover, autophagic flow was reduced by >50% in *SLC9A6* knockout neurons compared with wild-type neurons (17–19). Membrane trafficking from recycling endosomes is essential for the growth and maintenance of dendritic spines in long-term potentiation, which is thought to be the molecular basis of learning and memory (20). Thus, mutations in *SLC9A6* likely affect long-term potentiation. The female *SLC9A6* mutation carrier phenotypic spectrum encompasses learning difficulties, speech and language delay, mild-to-moderate intellectual disability, and behavioral issues. Consistent with many X-linked disorders, skewed X-inactivation may play a role in the clinical presentation of *SLC9A6* mutations in female carriers (6). Sikora et al. created a heterozygous *SLC9A6* knockout female mouse that expressed the mutant *SLC9A6* allele to follow the same neuroanatomic distribution and cell-specific patterns as hemizygous mutant males (21). The heterozygous knockout female mice developed progressive behavioral and neuropathological abnormalities that were similar to but milder than hemizygous male knockout mice.

Nearly 98.86% of patients with CS have epilepsy. Their EEG background rhythms were normal or abnormal with fast waves, and interictal epileptiform discharges were focal, multifocal, or widespread (7). Mathieu et al. found that electrical status epilepticus during sleep is a constituent feature of patients with CS (22), especially in the case of intellectual disability, autism, and severe speech delay, which may contribute to the diagnosis of CS (23). A detailed analysis of the clinical data of 53 patients with epilepsy-related conditions revealed that seizures started earlier than 3 years of age, with tonic–clonic episodes in nearly all cases. Other common seizure types included atypical absence, tonic seizures, myoclonic seizures, and focal onset seizures. Some patients were diagnosed with Lennox–Gastaut syndrome and West syndrome (Supplementary Table 4). These patients had poor outcomes despite treatment with multiple antiseizure medications, although VPA, LEV, clonazepam, and lamotrigine were more effective and more frequently used. Developmental regression after onset of seizure clusters was reported in some cases (2, 3, 24, 25). Ikeda et al. reported a case of developmental regression at the age of 3 years following resistance to antiseizure medication (24). We speculate that this resulted from progressive brain dysfunction due to uncontrollable and frequent seizures. The patient in this study had an acute onset of seizures at the age of 1 year and 3 months; the seizures had a fluctuating course and occurred in clustered outbreaks. The background rhythm of VEEG in this case was normal with multifocal spike and wave complexes and sharp waves, which was consistent with the reported cases. Before the onset of CS, the patient could stand alone, walk with assistance, and pinch objects with the thumb and index finger and had recognition performance. After the onset of CS, the patient exhibited developmental regression and could not sit up, crawl, or stand unaided. He received treatment with phenobarbital, diazepam, and OXC, which did not improve his condition.

Nearly 35.09% of patients with CS showed cerebellar atrophy on MRI. The hemisphere and cerebellar vermis were the most frequently affected areas. Bilateral temporal and occipital subarachnoid widening have been reported in several cases. Neuropathological studies have shown that cerebellar atrophy was caused by neuronal loss of Purkinje cells, and extensive progressive loss of Purkinje cells and gelatinization were observed in the cerebellum of *SLC9A6* mutant mice (11). For the patient in this study, a brain MRI performed at the age of 1 year and 2 months showed a prominent cisterna magna. The patient will be regularly followed-up with MRI. Additionally, the patient had PVCs and ventricular parasystole; however, arrhythmias have not been reported in patients with CS. We hypothesize that the arrhythmia could be related to the myocarditis lesions in the apical and basal segments of the left ventricle, and further investigation is required.

There are no effective treatments for CS. Antiseizure medications can be administered to patients with CS who have epileptic seizures according to clinical manifestations, and adjusted based on the therapeutic effect. Medically intractable epilepsy can be managed with a ketogenic diet, and rehabilitation training is available for patients with psychomotor retardation. There are two future directions for CS treatment: gene therapy and treatment according to CS pathogenesis. Ouyang et al. demonstrated that loss of NHE6 results in the over-acidification of the endosomal compartment and attenuates tropomyosin receptor kinase B (TrkB) signaling; mouse brains with disrupted NHE6 displayed reduced axonal and dendritic branching, synapse number, and circuit strength (1). Functional defects associated with TrkB signal attenuation due to NHE6 deficiency can be rescued by high levels of exogenous TrkB (26). The reported cases indicate a link between phenotype and clinical outcome in CS; patients with developmental regression, frequent uncontrollable seizures, and complicated abnormalities of other systems, such as the digestive and respiratory systems, have limited life expectancy. Further studies must verify this association and clarify its mechanistic basis.

## 5. Conclusion

This study reported a case of CS in a Chinese family and identified a *de novo* splice variant in intron 11 (c.1366 + 1G > C) of *SLC9A6* by WES. Relevant literature was summarized and clinical phenotypes of CS were analyzed. The results of this study expand the mutation spectrum of CS.

## Data availability statement

The datasets presented in this article are not readily available because of ethical and privacy restrictions. Requests to access the datasets should be directed to the corresponding author.

## Ethics statement

This study was approved by the Ethics Committee of The Third Affiliated Hospital of Zhengzhou University (no. 2021-062-01). Written informed consent from the patients/participants or patients/participants’ legal guardian/next of kin was not required to participate in this study in accordance with the national legislation and the institutional requirements. Written informed consent was obtained from the minor(s)’ legal guardian/next of kin for the publication of any potentially identifiable images or data included in this article.

## Author contributions

YD made substantial contributions to the conception and design of this study and agreed to be accountable for all aspects of the work in ensuring that questions related to the accuracy or integrity of any part of the work were appropriately investigated and resolved. RL participated in drafting the manuscript and in acquiring, collecting, analyzing, and interpreting the data. LJ participated in revising the manuscript. SZ, WT, LW, and ML participated in collecting and interpreting the data. TJ, XC, and SC participated in editing final draft and confirmed the authenticity of the data. All authors contributed to the article and approved the submitted version.

## Funding

Funding was received from the Open Research of Henan Key Laboratory of Child Brain Injury and Henan Pediatric Clinical Research Center (Reference: KFKT2021003), Open Project of the Children’s Neural Development Engineering Research Center of Henan Province (Reference: SG201910), and the Key Project of Medical Science and Technology of Henan Province (Reference: LHGJ20190339).

## Conflict of interest

The authors declare that the research was conducted in the absence of any commercial or financial relationships that could be construed as a potential conflict of interest.

## Publisher’s note

All claims expressed in this article are solely those of the authors and do not necessarily represent those of their affiliated organizations, or those of the publisher, the editors and the reviewers. Any product that may be evaluated in this article, or claim that may be made by its manufacturer, is not guaranteed or endorsed by the publisher.

## Footnotes

^1^https://web.expasy.org/translate/

^2^https://pubmed.ncbi.nlm.nih.gov/

^3^https://rarediseases.info.nih.gov/

^4^https://ghr.nlm.nih.gov/

^5^https://www.ncbi.nlm.nih.gov/books/NBK1116

^6^https://www.yiigle.com/index
