# Supplementary material for: Clinical and genetic analysis of Christianson syndrome caused by variant of SLC9A6: case report and literature review
Source: Front Neurol. 2023 May 5;14:1152696. doi: 10.3389/fneur.2023.1152696 (PMC10196350; doi:10.3389/fneur.2023.1152696)
Supplement: Supplementary file 1 [file Table_1.docx]

Supplementary Material

Clinical and genetic analysis of Christianson syndrome caused by a variant of the *SLC9A6* gene: A case report and literature review

Yan Dong^1,2*^, Ruofei Lian^1^, Liang Jin^1^, Shichao Zhao^1^, Wenpeng Tao^1^, Lijun Wang^1^, Mengchun Li^1^, Tianming Jia^1^, Xuejing Chen^1^, Shushi Cao^1^

*** Correspondence:** Yan Dong: yjs6690@126.com

# Supplementary Tables

**Supplementary Table 1** The system and conditions of PCR

1. PCR reaction system

| *TaKaRa Ex Taq* (5U/μl) | 0.25 μl |
| --- | --- |
| 10×*Ex Taq* Buffer (Mg^2+^ plus) (20mM) | 5 μl |
| dNTP Mixture (2.5mM each) | 4 μl |
| Template | ＜500 ng |
| Upstream primer | 0.2-1.0 μM (final conc.) |
| Downstream primer | 0.2-1.0 μM (final conc.) |
| Sterilized water | up to 50 μl |

1. PCR conditions

| Steps | Temperature (℃) | Time | Number of cycles |
| --- | --- | --- | --- |
| 1 | 98 | 10S | 35 |
| 2 | 98 | 10S |  |
|  | 58 | 30S |  |
|  | 72 | 60S |  |
| 3 | 72 | 10min |  |

**Supplementary Table 2** Primer sequence information related to Minigene experiment

| Subjects | Sequence information |
| --- | --- |
| SLC9A6-AF | 5’ - AAGCTTGGTACCGAGCTCGGATCCTTGTTTGAGCTTCTCAATTTCTTGGCAG - 3’ |
| SLC9A6-AR | 5’ - TTAAAAGCAGGCCTTCTGCCCTGTGGCATAGACTGC - 3’ |
| SLC9A6-BF | 5’ - GGCAGAAGGCCTGCTTTTAAACGTGTTTCTCTTAATC - 3’ |
| SLC9A6-BR | 5’ - TTAAACGGGCCCTCTAGACTCGAGCTGATATGCAAGCATGACAGCATTGCAG - 3’ |
| SLC9A6-MUT-F | 5’ - GTTTGCTGCTAAGTTGTAACTTTCTTCTCTTGCCCACTTC - 3’ |
| SLC9A6-MUT-R | 5’ - ACAACTTAGCAGCAAACATCATCATGTGTTGAAAA - 3’ |
| MiniRT-F | 5’ - GGCTAACTAGAGAACCCACTGCTTA - 3’ |
| SLC9A6-RT-R | 5’ - CTGATATGCAAGCATGACAGCATTG - 3’ |

# Supplementary Table 3 Summary of phenotypes in families with *SLC9A6* mutations

| **Reference** | **Number of patients/ families** | **Variant** | **Inherited/ de novo** | **ID/DD** | **Developmental regression** | **Autistic behavior** | **Verbal language absent** | **Epilepsy** | **Microcephaly** | **Ataxia** | **ophthalmoplegia** | **Cerebellar atrophy** | **Hyperkinesia** | **Sleep disorders** | **Angelman syndrome-like symptoms** | **Neuroimaging performed** |
| --- | --- | --- | --- | --- | --- | --- | --- | --- | --- | --- | --- | --- | --- | --- | --- | --- |
| Christianson et al. (1999) | 16/1 | p.H171fs  c.512_513del | Inherited (16/16) | 16/16 | 3/4 | 2/2 | 16/16 | 14/14 | 3/4 | 3/3 | 6/6 | 2/4 | − | − | − | Patients had cerebellar and brainstem atrophy. The cerebellum, most notably the vermis, was small and atrophic. |
| Gilfillan et al. (2008) | 8/3 | p.E255_S256del  c.764_769del  p.R468X  c.1402C>T  p.V144_R169del  c.507+ 1del | Inherited (8/8) | 7/7 | − | − | 7/7 | 7/7 | 7/7 | 7/7 | − | 1/3 | 5/7 | 3/7 | 7/7 | The Norwegian patient had progressive cerebellar atrophy. |
| Fichou et al. (2009) | 1/1 | p.A9S  c.25G>T | Inherited (1/1) | 1/1 | − | − | 1/1 | − | 1/1 | − | − | − | 1/1 | − | − | Normal |
| Garbern et al. (2010) | 6/1 | p.W338_T340del  c.1012_1020del | Inherited (6/6) | 6/6 | 3/6 | 6/6 | 4/6 | 5/6 | 2/6 | 6/6 | 5/6 | 2/6 | − | − | − | Axial T2-weighted imaging showed moderate ventricular enlargement and sulcal widening with normal appearance of white matter. A T1 sagittal slice through the midline showed a thin corpus callosum and moderate sulcal prominence and ventricular dilatation. |
| Schroeret al. (2010) | 7/2 | p.R468X  c.1498C>T  p.Q407X  c.1219C>T | Inherited (6/7)  De novo (1/7) | 7/7 | 4/7 | − | 7/7 | 7/7 | 6/7 | 1/7 | 6/6 | 3/7 | 1/7 | − | 3/5 | Family 1: Imaging findings showed focal atrophy and/or hypoplasia of the inferior cerebellar vermis with an associated subtle high signal in the inferior aspect of the cerebellar hemispheres on coronal FLAIR sequences. The cerebellar atrophy and/or hypoplasia was present at a similar location but was more conspicuous in the oldest subject.  Family 2: Brain MRI showed mild cerebellar volume loss involving the vermis and inferior aspects of the cerebellum. |
| Tzschach et al. (2011) | 1/1 | 314kb deletion in Xq26.3(SLC9A6 exons 15 and 16) | De novo | 1/1 | − | − | 1/1 | 1/1 | 0/1 | 1/1 | − | 0/1 | − | − | 0/1 | MRI revealed a small lipoma between the inferior colliculi of the tectum but no malformations or other abnormalities. |
| Takahashi et al. (2011) | 1/1 | p.S147fs  c.441delG | Inherited | 1/1 | 1/1 | − | 1/1 | 1/1 | 1/1 | 1/1 | 1/1 | 0/1 | 1/1 | − | 1/1 | − |
| Mignot et al. (2013) | 1/1 | p.G306X  c.916C>T | − | 1/1 | 1/1 | 1/1 | 1/1 | 1/1 | 1/1 | 1/1 | 1/1 | 1/1 | 1/1 | 1/1 | 1/1 | Cerebellar atrophy |
| Riess et al. (2013) | 4/2 | p.T489YfsX23  c.1464_1465insT  c.584 + 1 G > T | Inherited (4/4) | 4/4 | − | − | 1/1 | 4/4 | 4/4 | − | 1/1 | − | − | − | − | − |
| Schuurs-Hoeijmakers et al. (2013) | 2/1 | p. E547*  c.1639G>T | Inherited (2/2) | 2/2 | − | − | − | 2/2 | 2/2 | 2/2 | − | − | − | − | 2/2 | − |
| Bosemaniet al. (2014) | 2/2 | c.526+1G > A | De novo (2/2) | 2/2 | 0/2 | 1/2 | 1/2 | 2/2 | 2/2 | 2/2 | − | 2/2 | 1/2 | − | 1/2 | Patient 1: Head CT showed enlarged interfolial cerebellar spaces compatible with cerebellar atrophy. Brain MRI revealed atrophy of the vermis and cerebellar hemispheres (inferior parts more affected), diffuse FLAIR hyperintensity of the cerebellar cortex, and secondary enlargement of the fourth ventricle. The brainstem, basal ganglia, and supratentorial brain structures were within normal limits.  Patient 2: Brain MRI showed atrophy of the vermis and cerebellar hemispheres (inferior parts more severely affected), a patchy FLAIR hyperintense signal in the cerebellar cortex, and enlargement of the fourth ventricle and supravermian cistern. The brainstem, basal ganglia, and supratentorial brain structures were within normal limits. |
| Pescosolido et al. (2014) | 14/12 | p.G383D  c.1148G > A  p.R472fsX4  c.1414dupA  p.W570X  c.1710G > A  p.W523X  c.1568G > A  p.F183fsX1  c.540-547dup  p.E64X  c.190G > T  p.R500X  c.1498C > T  exons 10-16 deletion  IVS c.1237-557_UTR del  p.E547X  c.1639G> T | De novo (7/14)  Inherited (7/14) | 14/14 | 7/14 | 12/13 | 13/14 | 12/12 | 12/13 | 13/13 | 11/14 | 3/9 | 14/14 | 9/14 | 14/14 | There were 3 participants (33%) with documented cerebellar atrophy. MRI studies at sequential time points showed one patient with moderate-to-severe atrophy of the cerebellar hemispheres and vermis, associated with developmental regression (loss of the ability to walk). Another patient had bilateral lesions in the inferior cerebellum with minimal volume loss. There were also notable findings such as increases in ventricle size as well as changes in white matter. |
| Zanni et al. (2014) | 1/1 | c.1151-1G>A | De novo (1/1) | 1/1 | 1/1 | 1/1 | 1/1 | 1/1 | 1/1 | 1/1 | − | 1/1 | − | − | − | Neuroimaging revealed cerebellar atrophy affecting mostly the vermis, cerebral atrophy (operculum), enlarged ventricles, and reduced hippocampus size. |
| Coorg etal. (2015) | 1/1 | p.W570X  c.1710G>A | Inherited (1/1) | 1/1 | 0/1 | 1/1 | 1/1 | 1/1 | 1/1 | 0/1 | 0/1 | 0/1 | 0/1 | 1/1 | − | Normal |
| Masurel-Paulet et al. (2016) | 1/1 | Skipping of exon 3 (mRNA validation)  c.526-9_526-5del | Inherited (1/1) | 1/1 | 0/1 | 0/1 | 1/1 | 1/1 | 1/1 | 0/1 | 0/1 | 1/1 | 1/1 | − | − | Brain MRI revealed mild cerebellar atrophy. Mid-sagittal and coronal T2-weighted imaging showed focal lobular hypoplasia of tuber, pyramid, and uvula of the vermis and superior and inferior semilunar lobules of both cerebellar hemispheres. Hypoplasia of the vermis was associated with discrete global vermis atrophy. |
| Trump et al. (2016) | 2/2 | p.H203Lfs*10  c.608del  p. H408Nfs*2  c.1222_1226del | De novo (2/2) | 2/2 | − | − | − | 2/2 | − | − | − | − | 1/2 | − | − | − |
| Padmanabha et al. (2017) | 1/1 | p.E559K  c.1675G>A | Inherited (1/1) | 1/1 | 1/1 | 0/1 | 0/1 | 1/1 | 0/1 | − | − | 1/1 | − | − | − | Progressive diffuse cerebral and cerebellar atrophy |
| Fung et al. (2017) | 2/2 | c.794-2A>G  p. L280Afs*17  c.838_839 delinsG | − | 2/2 | − | 2/2 | − | 2/2 | 2/2 | − | − | − | − | − | − | − |
| Mathieu et al. (2018) | 5/3 | 40Mb deletion in Xq26.3  removed 9 exons  p.W523*  c.1569G > A  p.G383D  c.1148G > A | Inherited (3/5)  De novo (2/5) | 5/5 | 2/5 | 5/5 | 5/5 | 5/5 | 1/5 | 3/5 | 2/5 | 0/5 | 5/5 | 2/5 | 2/5 | Family 1: Slight enlargement of subarachnoid spaces, mostly in bitemporal regions, and a left temporal arachnoid cyst  Family 2: Normal  Family 3: Normal |
| Liu et al. (2018) | 1/1 | p.Y194fs  c.582_595del | − | 1/1 | − | − | − | 1/1 | − | − | − | 0/1 | − | − |  | − |
| Ilie et al. (2019) | 1/1 | p.G218R  c.1412 T > C | De novo (1/1) | 1/1 | 0/1 | 1/1 | 1/1 | 1/1 | 1/1 | 1/1 | 1/1 | − | 1/1 | 1/1 | 1/1 | − |
| Ieda et al. (2019) | 1/1 | multiple aberrant transcripts (mRNA validation)  c.1141-8C >A | De novo (1/1) | 1/1 | 1/1 | − | 1/1 | 1/1 | 1/1 | 1/1 | − | 0/1 | − | − | 1/1 | Normal |
| Ikeda et al. (2020) | 2/2 | p.R468 *  c. 1179C > T  p.I160Lfs*5  c.477_481del | Inherited (1/1)  De novo (1/1) | 2/2 | 1/2 | − | 2/2 | 2/2 | 2/2 | 2/2 | − | 2/2 | 1/2 | − | − | Patient 1: Mild atrophy in the cerebrum and cerebellum  Patient 2: T2 hyperintensity and atrophy of the lower cerebellum |
| Ibarluzea et al. (2020) | 1/1 | p.M106V  c.316A>G | Inherited (1/1) | 1/1 | − | − | − | − | − | − | − | − | − | − | − | − |
| Zhang et al. (2020) | 1/1 | Skipping of exon 6 (mRNA validation)  c.899+3_899+6del | De novo (1/1) | 1/1 | − | − | − | 1/1 | 1/1 | − | − | 0/1 | − | − | − | Normal |
| Yalcintepe et al. (2021) | 1/1 | p.T504LfsX8  c.1505_1509dup | Inherited (1/1) | 1/1 | 0/1 | 0/1 | 1/1 | 1/1 | 1/1 | 0/1 | 0/1 | 0/1 | 0/1 | 0/1 | 0/1 | Normal |
| Lan et al. (2021) | 1/1 | p.L517fs*5  c.1548_1549insT | De novo (1/1) | 1/1 | 0/1 | 0/1 | 1/1 | 1/1 | 1/1 | 0/1 | 0/1 | 0/1 | 0/1 | 0/1 | 1/1 | Normal |
| Liu et al. (2021) | 1/1 | p.L381_F418del  c.1237-2 A>G | Inherited (1/1) | 1/1 | 0/1 | 0/1 | 1/1 | 1/1 | 1/1 | 1/1 | 1/1 | 0/1 | 1/1 | 0/1 | 1/1 | MRI showed widening of the extracerebral space at bilateral temporal poles. |
| Gong et al. (2021) | 1/1 | p.X394del  c.1178_1180del | − | 1/1 | − | − | 1/1 | 1/1 | − | 1/1 | − | − | 1/1 | − | 0/1 | − |
| Zhang et al. (2022) | 1/1 | Skipping of exon 12 (mRNA validation)  c.1463-1G>A | De novo (1/1) | 1/1 | 0/1 | 0/1 | 1/1 | 1/1 | 1/1 | 1/1 | 0/1 | 1/1 | 1/1 | 0/1 | 0/1 | Dysplasia of inferior cerebellar vermis and fourth ventricle enlargement |
| Petraitytė et al. (2022) | 2/1 | c.899 + 1G > A | Inherited (2/2) | 2/2 | 0/2 | 1/2 | 1/2 | 2/2 | 2/2 | 1/2 | 0/2 | 0/2 | 1/2 | 0/2 | 0/2 | Patient 1: Head CT showed signs of partial dysplasia of the temporal lobe. |
| Mir et al. (2022) | 3/3 | p.L584F  c.1752G>T  c.680+3A>G  p.A532Gfs*9  c.1595_1613del | − | 3/3 | − | 1/3 | 3/3 | 3/3 | 2/3 | − | − | − | 2/3 | − | − | MRI showed bilateral symmetrical hyperintensities in occipital and cerebellar lobes. |
| Li et al. (2022) | 1/1 | p.R206*  c.616C>T | Inherited (1/1) | 1/1 | 0/1 | 0/1 | 0/1 | 1/1 | 0/1 | 1/1 | 0/1 | 0/1 | 1/1 | − | 0/1 | Bilateral temporal pole subarachnoid widening with no significant abnormal signal in the brain parenchyma |
| Song et al. (2022) | 1/1 | c.803+1 G>A | Inherited (1/1) | 1/1 | − | 1/1 | 1/1 | 1/1 | 1/1 | 0/1 | − | 0/1 | − | − | 0/1 | Normal |
| Chen et al. (2022) | 1/1 | p.P189L  c.566C>T | − | 1/1 | − | − | 1/1 | 1/1 | 1/1 | − | − | 0/1 | − | − | 0/1 | Normal |

Abbreviations: CT, computed tomography; DD, developmental delay; FLAIR, fluid attenuated inversion recovery; ID, intellectual disability; MRI, magnetic resonance imaging.

The symbol “−” indicates not mentioned.

# Supplementary Table 4 Epilepsy phenotype in families with *SLC9A6* mutation

| **Reference** | **Age of onset** | **Seizure type** | **ASMs** | **Treatment outcome** | **SE** | **Developmental regression after seizures** | **Electroencephalography** |
| --- | --- | --- | --- | --- | --- | --- | --- |
| Christianson et al. (1999) | Under 1 year | GTCS | CBZ | Effective |  |  | Recorded irregularities suggestive of epilepsy |
|  | Under 1 year | GTCS | CBZ | Effective |  |  | Exhibited diffuse epileptiform dysfunction with left frontotemporal focal features and a mild degree of diffuse slowing |
|  | 1 Year | GTCS | PB | Ineffective |  |  | − |
|  | 1 Year | GTCS | PHT, PB | − |  |  | Presence of sharp transients and an excess of diffuse slow activity. The dominant background frequency was 4–7 Hz. |
| Gilfillan et al. (2008) | 16 Months | GTCS, tonic seizure, CPS | − | − |  |  | Epileptiform activity, background frequency of 1.5–3 Hz |
| Schroer et al. (2010) | 23 Months | GTCS | PB | Effective |  |  | − |
|  | Under 2 years |  |  |  |  |  | − |
|  | 7 Months | GTCS | PB, VPA, PHT | Ineffective; died of seizures at age 29 years | E |  | − |
|  | 2 Years | GTCS | PB, VPA, LEV |  |  |  | − |
|  | 15 Months | LGS, GTCS, AA, and atonic seizures between the ages of 6 and 7 years | − | − |  |  | Frontal high-amplitude 2–3-Hz rhythmic spike-wave activity in addition to other findings consistent with Lennox–Gastaut syndrome |
|  | 16 Months | GTC, AA | CBZ, LEV | Ineffective | E |  | − |
|  | 10 Months | GTCS, AA | AEDs, ketogenic diet, VNS, OXC, CZP | GTCS decreased significantly after VNS, OXC, CZP application, but AA became more frequent |  | E | Bifrontal dominance of multifocal spikes with burst activity and interictal discharges with a predominant alpha rhythm throughout the discharges |
| Tzschach et al. (2011) | 11 Months | − | CZP | Effective |  |  | Normal |
| Takahashi et al. (2011) | 4 Years | Various types | ACTH、AEDs | Ineffective |  | E | Background frequency of 5–6 Hz theta waves and spontaneous appearance of 3-Hz diffuse high-voltage slow waves |
| Mignot et al. (2013) | 2 Years | GTCS | AEDs | Effective |  |  | Disorganized background with bilateral frontal slow waves and spikes waves, and short subclinical generalized spike bursts |
| Riess et al. (2013) | 10 Months | − | − | − |  |  | − |
|  | 16 Months | − | − | − |  |  | − |
| Bosemani et al. (2014) | 2 Years | GTCS | RUF, CLB, LTG, ketogenic diet | Ineffective | E |  | − |
|  | 2 Years | − | AEDs | Ineffective |  |  | − |
| Pescosolido et al. (2014) | 4 Months to 3 years (mean age, 16.4±7.86 months) | Infantile spasms, tonic seizures, GTCS, MC, drop seizures (unknown whether tonic or atonic) and episodes described as staring spells, focal onset seizures. | − | − |  | Present in 50% of patients | Patient 1: 1, Generalized spike-wave activity from 1.5–4 Hz (at 2, 14, and 15 years); 2, Rhythmic theta activity, normal posterior dominant rhythm (8 Hz), absent sleep architecture (14 years)  Patient 2: Generalized epileptiform activity, further details unavailable (18 months and 3 years)  Patient 3: 1, Intermittent central sharp waves, normal posterior dominant rhythm (9 months); 2, Multifocal and bilateral synchronized epileptiform discharges clinically correlated with myoclonus, interictal generalized slow spike and wave discharges; 3, Normal EEG (9 years)  Patient 4: 1, Normal posterior dominant rhythm (8–9 Hz), bifrontal spikes, sleep potentiation (21 months); 2, High-voltage, intermittent, generalized fast spikes with no clinical correlates, normal posterior dominant rhythm (9–10 Hz) (7 years)  Patient 5: Bilateral, multifocal, and generalized epileptiform discharges (1, 2, and 4 years)  Patient 6: Near-continuous, generalized, high-voltage, semi-rhythmic to rhythmic, 2–4-Hz spike and slow-wave complexes, absence of normal background, no clinical changes during discharges  Patient 7: 1, Abnormal (1–10 years), no further data available; 2, Normal (>10 years)  Patient 8: Normal (parental reporting)  Patient 9: “Epileptic activity” (parental reporting)  Patient 10: No EEG  Patient 11: 1, Hypsarrhythmia (5 months); 2, Focal, frontal and right central epileptiform discharges with resolution of hypsarrhythmia (6 months); 3, Generalized rhythmic activity with anterior predominance (13 months); 4, High-voltage background with scattered sharp waves (20 months)  Patient 12: Frequent generalized slow spike-wave discharges  Patient 13: Frequent frontal spikes with a suppressed background (possible medication effect, <17 months) |
| Zanni et al. (2014) | Under 2 years | GTCS | AEDs | Ineffective |  | E | Awake EEG showed normal background activity with multiple superimposed spikes and spikes and waves over both frontal—temporal areas that were associated with more diffuse discharges of short duration (1–3 s) during up to 60% of wakefulness periods. During sleep, epileptiform abnormalities increased and became generalized. Continuous spike and wave activity was present during sleep, consistent with diagnostic criteria for ESES. |
| Coorg et al. (2015) | 1 Year | GTCS, MC, AA | CBZ, LEV, VPA | Ineffective | E |  | 12 Months: Diffuse generalized discharges that were more frequent during sleep  2 and 4 Years: A 9-Hz posterior dominant rhythm  4 Years: Mild diffuse slowing and bifrontal and occasional right central sharp waves  8 Years: Frontally predominant generalized spike and wave interictal epileptiform discharges during wakefulness and sleep; the generalized discharges constituted >85% of the slow wave sleep recording, a pattern consistent with ESES. |
| Masurel-Paulet et al. (2016) | 3 Years | GTCS | VPA | Effective |  |  | Normal |
| Padmanabha et al. (2017) | Under 1 year | Focal (right-sided), GTCS, epileptic spasms | − | − |  |  | Right hemispheric slowing with frequent bilateral frontotemporal interictal discharges |
| Mathieu et al. (2018) | 14 Months | FS, AA, MC, GTCS, atonic seizures | VPA, ESM, CLB | Ineffective |  |  | 9 Months: Normal background with intermixed diffuse bursts of spike and polyspike-waves, lasting not more than 2s.  32 Months: Abnormal fast-background rhythm, sleep rhythms were poorly differentiated but there was no paroxysmal activity  5 and 8 Years: Multifocal, mostly rolandic, independent spike-wave discharges that became more frequent and synchronous during sleep at the age of 8 years. Follow-up sleep EEG recordings could not be obtained due to agitation of the subject. |
|  | 13 Months | MC, GTCS | VPA, CLB, LEV, CZP, ESM, LTG | Effective |  |  | 12 Months: Abnormal fast 8–9 Hz background rhythm with poor anterior posterior organization and frontal or diffuse spikes and polyspike wave discharges enhanced during sleep  4 Years: Diffuse paroxysmal activity increased significantly during sleep  6.5 Years: Background rhythm decreased to 7–8 Hz, diffuse paroxysmal activity constituting 80% of the NREM sleep recording  8 Years: Background rhythm decreased to 4–5 Hz, intermixed with beta activity, consistent with diagnostic criteria for ESES. Interictal discharges progressively decreased and ceased at the age of 17 years. |
|  | 20 Months | GTCS | VPA, LEV, CLB | Ineffective | E |  | Initial EEG recordings showed abnormally fast background activity and bifrontal spikes that became subcontinuous and progressively more diffuse during sleep, eventually meeting the diagnostic criteria for ESES. |
|  | 17 Months | GTCS | LEV | Ineffective |  |  | 35 Months: Diffuse alpha (10 Hz) background activity and sporadic spikes |
|  | 22 Months | GTCS | VPA, LTG, OXC, CBZ | Ineffective |  |  | 17 Months: A 7-Hz background rhythm, frontal spikes and polyspike wave discharges enhanced during sleep  4 Years: ESES |
| Ikeda et al. (2020) | 9 Months | Tonic seizure, AA, LGS | VPA, CZP, TPM | Ineffective |  | E | 11 Months: Sleep EEG showed frontal and occipital spike–wave complexes and generalized slow spike–wave complexes  7 Years: Sleep EEG showed frequent generalized slow spike–wave complexes  12 Years: Generalized paroxysmal fast activity |
|  | 17 Months | Tonic seizure, AA, LGS | VPA, CLB, TPM, LTG, LEV | Ineffective |  |  | 3 Years: Frequent generalized slow spike–wave complexes  7 Years: ESES |
| Lan et al. (2021) | 13 Months | GTCS | − | − |  |  | − |
| Liu et al. (2021) | 11 Months | Tonic seizure, GTCS | VPA, LEV | Effective |  |  | 2 Years 10 months: Abnormal |
| Zhang et al. (2022) | 12 Months | GTCS, MC | TPM, VPA, LEV, rTMS, LTG, ZNS, OXC | Effective |  |  | Awake EEG revealed diffuse, irregular, medium-to-high–amplitude 4–6 Hz theta rhythm, middle-to-very high-amplitude spikes, multiple spikes, spike and slow wave complexes, and frequent and asynchronous rhythms. Frontal, occipital, and temporal leads were prominent, especially in the right hemisphere. Epileptiform discharges were more severe during sleep than during wakefulness. |
| Petraitytė et al. (2022) | 11 Months | − | − | − |  |  | Irregular delta-, alpha-, and theta-wave polymorphic activity during sleep |
|  | 1.5 Hours | GTCS | − | − |  |  | − |
| Li et al. (2022) | Under 2 years | AA | VPA, LEV, PB, TPM, LTG, MPPT | Effective |  |  | Slightly slow background, multifocal spikes and spike slow waves predominantly in the frontotemporal area bilaterally or widespread during wakefulness and sleep periods; the discharges constituted 75% of the NREM sleep recordings.  After MPPT at the age of 7 years: Multifocal spikes and spike slow waves during sleep |
| Song et al. (2022) | 11 Months | Generalized seizures | LEV | Ineffective |  |  | Spike slow waves were distributed in the central midline area |
|  | 40 Months | GTCS | VPA | Effective |  |  | Spikes, spike slow waves, and polyspike slow waves were distributed in the rolandic area bilaterally |
| Chen et al. (2022) | 4 Months | GTCS, epileptic spasms | VPA, ACTH, TPM | Ineffective |  |  | High- and extremely high-amplitude spikes and sharp waves mixed with slow waves or short-range bursts during sleep; a few highly arrhythmic patterns appeared intermittently during sleep |

Abbreviations: AA, atypical absence; ASMs, antiseizure medications; CBZ, carbamazepine; CLB, clobazam; CPS, complex partial seizure; CZP, clonazepam; E, the patient had this clinical presentation; EEG, electroencephalogram; ESES, electrical status epilepticus during sleep; ESM, ethosuximide; FS, febrile seizures; GTCS, generalized tonic-clonic seizure; LEV, levetiracetam; LGS, Lennox-Gastaut syndrome; LTG, lamotrigine; MC, myoclonic seizures; MPPT, methylprednisolone pulse treatment; NREM, non-rapid eye movement; OXC, oxcarbazepine; PB, phenobarbitone; PHT, phenytoin; rTMS, repetitive transcranial magnetic stimulation; RUF, lufilamide; SE, status epilepticus; TPM, topiramate; VGB, vigabatrin; VNS, vagus nerve stimulation; VPA, valproate; ZNS, zonisamide.

The symbol “−” indicates not mentioned.
